# Theobromine versus casein phospho-peptides/Amorphous calcium phosphate with fluoride as remineralizing agents: effect on resin-dentine bond strength, microhardness, and morphology of dentine

**DOI:** 10.1186/s12903-023-03139-z

**Published:** 2023-07-04

**Authors:** Lamia M. Elmalawany, Dalia I. Sherief, Ghada A. Alian

**Affiliations:** Biomaterials Department, Faculty of Dentistry, Ain-Shams University, African union organization street, 11566 Abbasia, Cairo Egypt

**Keywords:** Remineralization, MI paste plus, Theobromine, Bond strength, Microhardness

## Abstract

**Background:**

This study aimed to assess the impact of theobromine and casein phospho-peptides/amorphous calcium phosphate with fluoride (CPP-ACPF) on the resin-dentine bond strength, microhardness, and dentine morphology.

**Methods:**

A total of 18 sound human molars for micro-tensile bond strength (µTBS), 20 sound human premolars for microhardness, and 30 premolars for Scanning electron microscopy/energy-dispersive X-ray spectroscopy (SEM/EDX) were used. Based on the pre-treatment used, teeth were split into six groups; sound dentine, demineralized dentine, and demineralized dentine treated with theobromine (Sigma Aldrich) and MI paste plus (GC International, USA) for two-time intervals; 5 min and 1 month. The bonded teeth were sectioned to produce 1 mm^2^ resin-dentine sticks which were evaluated for µTBS using a universal testing device (Instron 3365, USA). The dentine microhardness was tested by using the Vickers microhardness tester (Nexus 4000 TM, Netherlands). The pre-treated dentine surface was examined using SEM/EDX (Neoscope JCM-6000 plus Joel benchtop SEM, Japan). µTBS results were analysed with two-way ANOVA. Microhardness and EDX results were analysed with two-way mixed model ANOVA. The significance level was set at (p ≤ 0.05).

**Results:**

While both remineralizing materials at the two-time intervals demonstrated µTBS comparable to sound dentine (46.38 ± 12.18), the demineralized group demonstrated statistically the lowest µTBS (p < 0.001). Whether used for 5 min or 1 month, theobromine significantly increased the microhardness (50.18 ± 3.43) and (54.12 ± 2.66) respectively (p < 0.001), whereas MI paste only increased the hardness (51.12 ± 1.45) after 1 month (p < 0.001).

**Conclusions:**

The pre-treatment of demineralized dentine with theobromine for 5 min or 1 month could enhance its bond strength and microhardness while for MI paste plus, only 1-month application was efficient to ensure remineralization.

## Background

Dental caries results from the improper balance between the demineralization caused by the action of bacterial acids and the remineralization rate in the tooth structure [1] leading to the loss of the mineralized tissue and the derangement of collagen [2].

The recent modalities in treating dental carious lesions involve the excavation of the soft superficial infected dentine while preserving the deeper re-mineralizable layer [3]. Thus, the tooth structure substrate for bonding to restorations is composed of both sound dentine on the axial walls and caries-affected dentine (CAD) on the floor. [2].

CAD is characterized by its decreased mechanical properties and altered morphological and chemical features compared to sound dentine [4], which irrelevant of the adhesive system applied, negatively compromises the bonding strength to composite resin [5–7], where cohesive failure in dentine was quite common [4, 8].

Remineralizing agents have reportedly been used on dentine to potentially restore its mechanical properties [9]. Multiple remineralizing agents are available, of which fluoride is the gold standard [10, 11]. Despite that, there have been concerns about their long-term use due to the fear of fluorosis and fluoride toxicity especially in children [12]. Fluoride can cross the cell membrane reaching a wide range of cell types and affecting their function. Fluoride toxicity can cause digestive system irritation and lower children’s IQ [11]. Moreover, the oral fluids’ content of calcium and phosphate ions affects fluoride’s capacity to reduce the occurrence of caries [13].

Dental researchers are constantly launching more biocompatible, natural anti-cariogenic materials to be used [13]. Due to its exceptional bioactivity related to the bioavailability of calcium and phosphorous in its composition, amorphous calcium phosphate (ACP), a precursor to hydroxyapatite (HA), has been employed as a dispersion phase in polymeric materials. However, their high solubility in aqueous solutions negatively affects their integrity in the oral cavity thus limiting their dental applications. New attempts have been done to make them metastable through the formation of a nano-complex of casein phospho-peptides/amorphous calcium phosphate (CPP-ACP) [2]. Casein phospho-peptides (CPP) a milk protein derivative that has a role in enamel and dentine remineralization; by releasing calcium and phosphate ions thus deactivating the demineralization and enzymatic degradation processes. CPP-ACP has been evaluated as a remineralizing agent on enamel and dentine in terms of microhardness testing where its use increased the microhardness of demineralized tissues [14–16]. The effect of its use on dentine bond strength has been evaluated, but the results were controversial [17, 18].

Another natural product is theobromine, a white crystalline powder, that is one of the methylxanthines extracted from cocoa beans. It has been evaluated in many studies as a remineralizing agent for enamel by increasing its crystallite size [19–21]. Theobromine-containing toothpaste was found to have dentinal tubules occluding ability when used for 1 week [22]. Moreover, theobromine has shown a significant antimicrobial effect and reduced *Streptococcus mutans* biofilm deposition [23, 24]. These results showed a promising effect of theobromine as a remineralizing agent to dentine but to the best of our understanding, it has not yet been determined how its use would affect resin-dentine bond strength and microhardness.

So, this study aimed to investigate the effects of theobromine and MI paste plus (CPP-ACP with fluoride) application for 5 min and 1 month on the surface morphology, microhardness, and resin-dentine bond strength of demineralized dentine.

The null hypothesis tested was that the surface morphology, microhardness, and resin-dentine bond strength of demineralized dentine will not be impacted by the application of either remineralizing agent for different time intervals.

## Methods

### Materials

Materials utilized in this study, their description, composition, manufacturer, and lot numbers are presented in Table 1.

**Table 1:** Materials utilized in this study their description, composition, manufacturer, and lot numbers

Material	Description	Composition	Manufacturer	Lot Number
All Bond Universal	Ultra-mild Universal adhesive (pH > 3)	10-MDP, Bis-GMA, HEMA, Ethanol, Water, Initiators	Bisco, Schaumburg, Illinois, USA	2,100,006,253
Grandio	Universal nanohybrid resin composite.	Bis-GMA, TEDGMA, UDMA matrix. 87 wt% / 71.4 Vol.% inorganic filler loading.	VOCO GmbH, Cuxhaven, Germany	2,050,486
Meta Etchant	Etchant delivery system	37% Phosphoric Acid Semi Gel	META BIOMED CO.LTD., Korea	MET2009121
Han temp	Light curing temporary filling material.	UDMA, HEMA, Polyacrylic acid, Silicon dioxide	HANDAE CHEMICAL CO., LTD, Korea	HT0762010
Theobromine ≥ 98.0%	Cocoa Extract	3,7-Dimethylxanthine,3,7-Dihydro-3,7-dimethyl-1 H-purine-2,6-dione, 2,6-Dihydroxy-3,7-dimethyl purine	Sigma Aldrich	BCBZ9934
MI paste plus	Topical Tooth Créme Containing Calcium, Phosphate & Fluoride	NaF (900 ppm F) 0.2% wt/vol, CPP-ACP 10% wt/vol Water, Glycerol, D-sorbitol, CMC-Na, Propylene glycol, Silicon dioxide, Titanium dioxide, Xylitol, Phosphoric acid 75%, Sodium saccharin, Flavor, Ethyl Para hydroxybenzoate, Propyl Para hydroxybenzoate, Butyl Para hydroxybenzoate	GC International, USA	201112D

### Sample size calculation

The sample size for all tests was calculated using G power software with a power of 80%. For µTBS, according to a pilot study, a total of 120 samples subdivided into 20 samples for each experimental group was sufficient. For microhardness, based on the results of a previous study [25], the sample size was identified to be 16 samples subdivided into 8 samples for each material but ten samples per group were prepared (n = 10) to compensate for laboratory errors. For EDX, based on the results of a previous study [26], the sample size was identified to be 18 samples subdivided into 9 samples for each material.

### Micro-tensile bond strength (µTBS)

Eighteen sound human third molars recently extracted within the 20- to 45-year age range were collected, ultrasonically cleaned, rinsed, and kept in 0.1% thymol at 4 °C for no more than two months [17].

With a diamond saw (Isomet 4000, Buehler, Lake Bluff, IL, USA), the occlusal surfaces of teeth were removed under water coolant to reveal a flat mid-dentine surface [27]. The root ends were sectioned, and rotary endo files were used to open the canals to allow the simulated body fluid (SBF) with pH adjusted to 7.4, (8.035 g NaCl, 0.355 g NaHCO_3_, 0.225 g KCl, 0.231 g K_2_HPO_4_.3H_2_O, 0.311 g MgCl_2_.6H_2_O, 39 ml 1.0 M-HCL, 0.292 g CaCl_2_, 0.072 g Na_2_SO_4_, 6.118 g Tris and 0–5 ml 1.0 M-HCL) [28] to reach the interface.

The occlusal surfaces of teeth were ground under water coolant with 600-grit grit silicon carbide paper for 1 min followed by water rinsing [29].

**Fig. 1:**
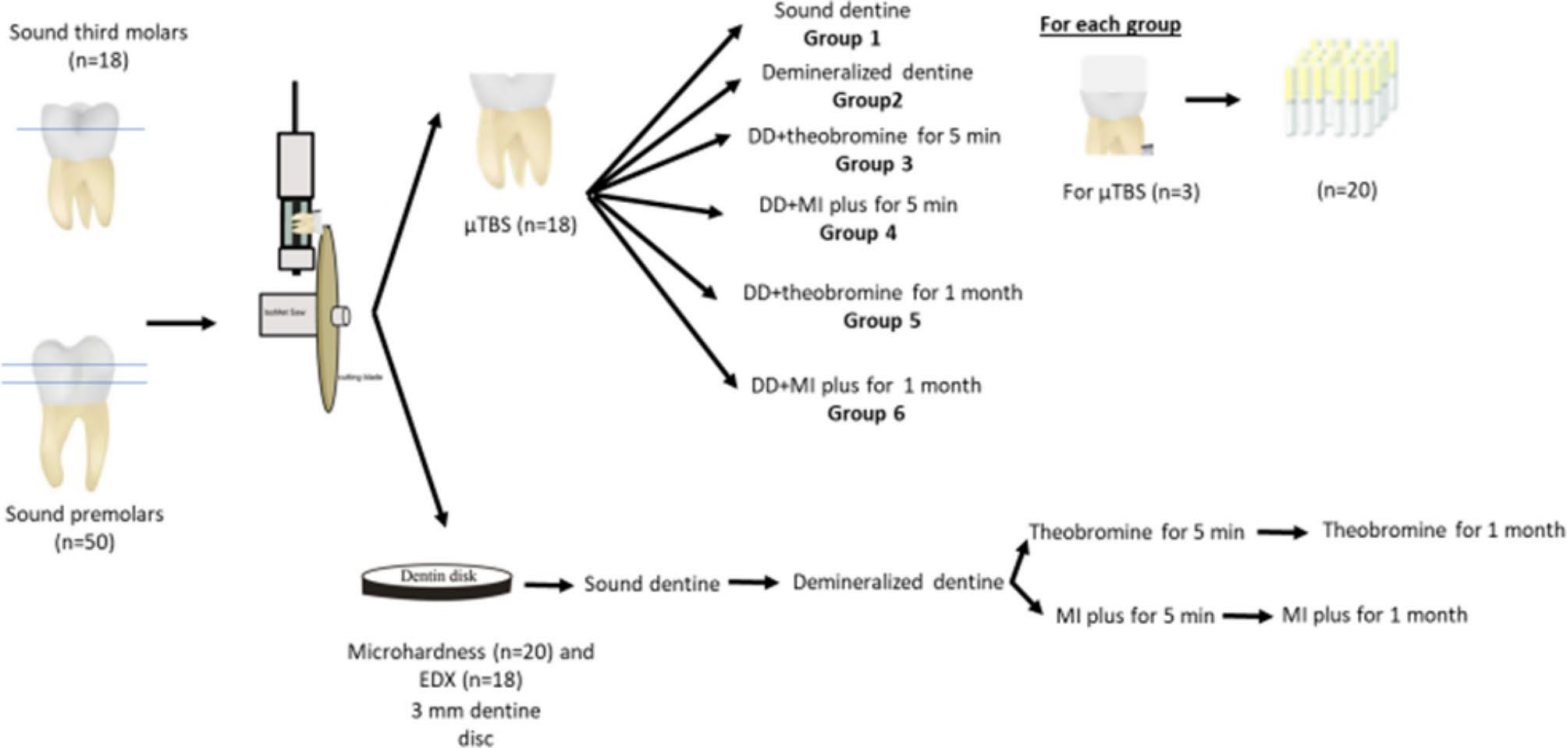
Flowchart summarizing the specimens’ grouping

As illustrated in Fig. 1, teeth were randomly distributed into 6 groups; Group I: sound dentine (positive control), Group II: demineralized dentine (negative control), Group III: demineralized dentine + 0.1 mL of the 200 mg/l theobromine solution (5 min), Group IV: demineralized dentine + 0.1 mL of the MI paste plus (5 min), Group V: demineralized dentine + 0.1 mL of the 200 mg/l theobromine solution (1 month) and Group VI: demineralized dentine + 0.1 mL of the MI paste plus (1 month).

A double coating of acid-resistant nail polish (Yolo cosmetics, Egypt) was applied to teeth (except group I), leaving only the dentine surface exposed [30]. Each tooth was placed inside a demineralizing solution-filled polyethylene tube (50 mM acetate buffer solution at pH 4.5, 2.2 mM KH_2_PO_4_, 0.5 ppm fluoride in the form of NaF, 2.2 mM CaCl_2,_ and 50 mM lactate gel) prepared according to Smith et al. [30] for 72 h at room temperature [31]. The volume of the solution used was equal to double the exposed dentine area [31]. After being removed from the demineralizing solution, the teeth were washed with deionized water for 15 min, ultrasonically cleaned, and gently dried.

For groups III & IV, the materials were applied using a micro-brush and allowed to sit for 5 min and any excess was wiped with absorbent paper. For groups V & VI, the materials were applied and then capped by a light-cured temporary material that was cured for 20s. Groups I, II, III, and IV were bonded immediately while groups V & VI were stored in SBF for 1 month at 37℃ in an incubator (Titanox, art.a3-213-400I, Italy) before bonding. The SBF was renewed daily.

Bisco all bond universal was used in etch-and-rinse mode. Enamel acid etching was performed using Meta acid etch for 15s then the remaining dentine surface for another 15s, after which the tooth was rinsed for 30s and dentine blot dried leaving the surface visibly moist. A contoured metal matrix with clamp no. 1552 (TOR VM, Russia) was used for the build-up of the resin composite.

The adhesive was applied using a micro-brush in 2 coats actively applied for 15s as recommended by manufacturer instructions and then air thinned till it no longer moved and light cured for 10s using LED light cure of 1200 mW/cm2 output and 10 mm in diameter tip (Elipar S10, 3 M ESPE, Germany). The composite build-up was done in 2 increments 2 mm each and light cured for 20s and the final increment was cured through a microscopic slide to avoid the oxygen-inhibited layer for 60s. The matrix was removed, and the resin composite was finished using finishing stones.

Isomet 4000 was used to serially section the teeth longitudinally perpendicular to the bonded interface in the x- and y-axes while using water cooling to produce resin-dentine sticks having a cross-sectional area of nearly 1 mm^2^ [32]. Each stick was individually attached using cyanoacrylate-based glue to a specially constructed attachment leaving the interface free [32] and tested in a universal testing device (Instron 3365, Norwood, MA, USA) at a cross-head speed of 1 mm/minute till debonding. Tensile stresses (MPa) were calculated using the following equation δ = Force/Area [27] using Bluehill 3 software.

After debonding each specimen, the interface was imaged using a stereomicroscope (Olympus Stereozoom SZ 40 Microscope, Tokyo, Japan) at magnification (X40) to determine the failure mode. Failure modes were categorized as adhesive (at the resin-dentine interface), cohesive (totally within resin composite or dentine substrate), or mixed (at the resin-dentine interface including one of the substrates).

### Dentine microhardness

With the same regulations as mentioned before, twenty sound human premolars extracted for orthodontic reasons were utilized. Isomet 4000 was used to obtain a mid-dentine disc of 3 mm thickness from each tooth.

A standard smear layer was created on the coronal side of dentine discs using 600-grit carbide papers followed by water rinsing for 1 min [25].

Microhardness measurements for the sound dentine were recorded using a digital Vickers hardness tester (Nexus 4000 TM, INNOVATEST, model no. 45.3, Netherlands). Three indentations 150 microns from the dentine-enamel junction were done for each specimen using a load of 200 gm and dwell time of 20 s, examined at magnification (X40), and the average of the three readings was calculated [33, 34].

Dentine discs were subjected to demineralization as mentioned before and the hardness testing was repeated (demineralized dentine).

After that, discs were randomly assigned into two groups (n = 10) depending on the remineralizing material utilized. The dentine surface was treated for 5 min with the remineralizing agent, followed by excess removal. They were placed in SBF for 24 h, after which the hardness testing was repeated.

Discs were then covered by a light-cured temporary material and stored for 1 month in SBF, after which the hardness testing was repeated once again. (Fig. 1)

### Scanning electronic microscope/ energy-dispersive X-ray spectroscopy (SEM/EDX)

Thirty sound human premolars were used, and dentine discs were prepared as mentioned for the microhardness testing. EDX (Neoscope JCM-6000 plus Joel benchtop SEM, Nikon, Japan) was employed to identify the changes in the Ca/P ratio on the surface of the various dentine groups. The specimens were prepared according to the already reported method in the microhardness testing using the same sample as a positive control, negative control, 5 min remineralization, and 1-month remineralization.

For the dentine surface morphology, twelve dentine discs were prepared [35] and randomly allocated into six groups as mentioned in the µTBS testing. (n = 2) SEM (Neoscope JCM-6000 plus Joel benchtop SEM, Nikon, Japan) was used to examine the specimens after they had been dried for 24 h at room temperature and sputter-coated with gold for 90 s at 15 mA (Hummer 8, Ladd Research, USA) at a standardized magnification (X1000) and (X3000).

### Statistical analysis

Numerical data were reported as mean and standard deviation values and were analysed for normality and variance homogeneity by utilising the Shapiro-Wilk and the Leven’s tests respectively. They demonstrated homogeneous variances and parametric distribution across groups. Two-way ANOVA was used to analyse the µTBS followed by Tukey’s post hoc test. Two-way mixed model ANOVA was used to analyse the microhardness and EDX followed by Tukey’s and the Bonferroni post hoc tests for independent and dependent variables respectively. The Bonferroni correction and pooled error terms from the primary ANOVA model were used to compare the main and simple effects. The significance level was set at (α = 0.05 within all tests). R statistical analysis software version 4.1.3 for Windows (R Core Team (2022). R: A language and environment for statistical computing. R Foundation for Statistical Computing, Vienna, Austria. URL https://www.R-project.org/) was utilised to perform the statistical analysis.

## Results

I. µ-TBS:

The mean and standard deviation (SD) values of µ-TBS (MPa) for different groups are presented in Table 2.

Group II (demineralized dentine) showed the lowest significant µ-TBS values compared to other groups with no significant difference between them.

**Table 2:** Mean ± standard deviation (SD values) of µ-TBS (MPa) values for different groups

Micro-tensile bond strength (MPa) (mean ± SD)						p-value
Sound dentine Group I	**Demineralized dentine** **Group II**	**Theobromine** **(5 min)** **Group III**	**MI paste plus** **(5 min)** **Group IV**	**Theobromine** **(1 month)** **Group V**	**MI paste plus** **(1 month)** **Group VI**	
46.38 ± 12.18^ A^	33.20 ± 7.80^B^	44.77 ± 10.30^ A^	43.77 ± 7.86^ A^	46.53 ± 8.63^ A^	46.80 ± 7.23^ A^	< 0.001*

Means with different superscript letters within the same horizontal row are statistically significantly different *; significant (p ≤ 0.05) ns; non-significant (p > 0.05)

II. Dentine microhardness:

The mean and standard deviation (SD) values of microhardness (VHN) are presented in Table 3.

Regarding the effect of the remineralizing materials, theobromine showed significantly higher microhardness values (50.18 ± 3.43) than MI paste plus (37.20 ± 0.33) (p < 0.001) after 5 min of remineralization while for the 1-month remineralization, there was no significant difference (p = 0.057) between MI paste plus (51.12 ± 1.45) and theobromine (54.12 ± 2.66).

Regarding the effect of time, theobromine showed no significant difference between the 5 min (50.18 ± 3.43) and 1-month remineralization (54.12 ± 2.66) values which was higher than the demineralized group (31.52 ± 1.78). MI paste plus showed no significant difference between the 5 min remineralization (37.20 ± 0.33) and the demineralized group (30.85 ± 6.28) while the 1-month remineralization showed significantly higher values (51.12 ± 1.45).

**Table 3:** Mean ± standard deviation (SD) values of microhardness and Ca/P ratios for different time intervals

		Theobromine	MI paste plus	p-value
Microhardness (VHN) (mean ± sd)	**Sound Dentine**	65.41 ± 4.25^ A^	65.95 ± 6.12^ A^	**0.875ns**
	**Demineralized Dentine**	31.52 ± 1.78^ C^	30.85 ± 6.28^ C^	**0.823ns**
	**5 min remineralizing agents**	50.18 ± 3.43^B^	37.20 ± 0.33^ C^	< 0.001*
	**1-month remineralizing agents**	54.12 ± 2.66^B^	51.12 ± 1.45^B^	**0.057ns**
	**p-value**	< 0.001*	< 0.001*	
Ca/P ratio (mean ± sd)	**Sound dentine**	1.69 ± 0.02^ A^	1.65 ± 0.03^ A^	**0.138ns**
	**Demineralized Dentine**	1.01 ± 0.09^B^	1.05 ± 0.28^B^	**0.804ns**
	**5 min remineralizing agents**	1.86 ± 0.03^ A^	1.20 ± 0.08^B^	< 0.001*
	**1-month remineralizing agents**	1.90 ± 0.01^ A^	1.68 ± 0.10^ A^	0.004*
	**p-value**	< 0.001*	< 0.001*	

Means with different superscript letters within the same vertical column are statistically significantly different *; significant (*p* ≤ 0.05) ns; non-significant (p > 0.05)

**Fig. 2:**
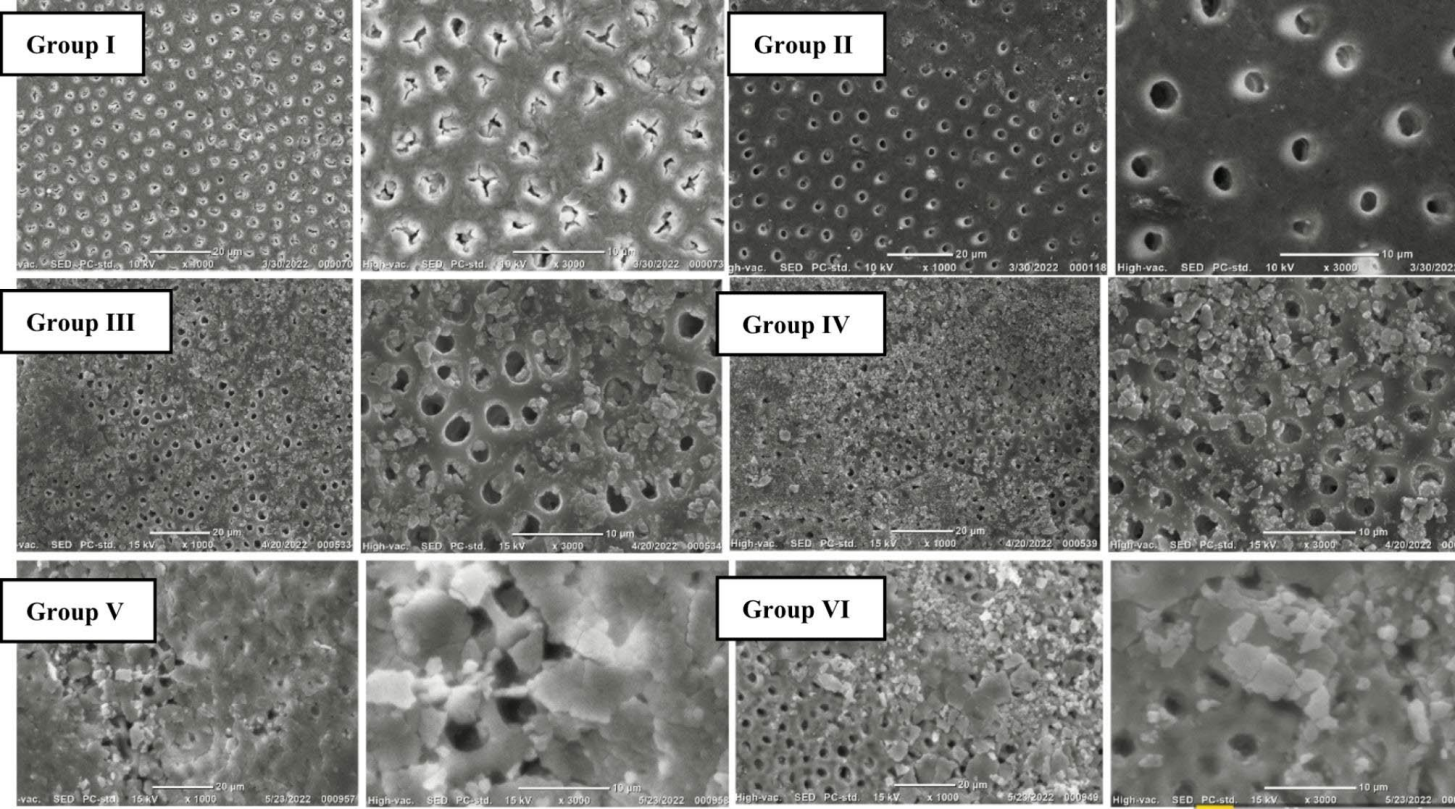
SEM images showing dentine surface morphology of the different groups at 1000X and 3000X magnification

III. SEM/EDX:

1. Dentine surface morphology (SEM):

Scanning electron micrographs showing the dentine surface morphology of all groups are presented in Fig. 2.

The dentinal tubules on the surface of sound dentine remained closed while those of the demineralized dentine were mostly open. After 5 min of application of theobromine and MI paste plus, only little minerals were deposited, and remineralization did not considerably obstruct the dentinal tubules but, theobromine showed more mineral deposits. After 1-month, more mineral deposits were observed on the dentine surface with heterogenous distribution; however, deposits did not sufficiently occlude the open dentinal tubules.

### 2. EDX

The mean and standard deviation (SD) values of the Ca/P ratio for different time intervals are presented in Table 3.

Regarding the effect of the remineralizing materials, theobromine (1.86 ± 0.03) (1.90 ± 0.01) had a significantly higher Ca/P ratio value than MI paste plus (1.20 ± 0.08) (1.68 ± 0.10) for the 5 min remineralizing and the 1-month respectively.

Regarding the effect of time, for theobromine, no significant difference was found between the 5 min (1.86 ± 0.03) and the 1-month remineralization (1.90 ± 0.01), which was higher than demineralized dentine (1.01 ± 0.09). For MI paste plus, no significant difference was found between the 5 min remineralization (1.20 ± 0.08) and the demineralized group (1.05 ± 0.28), and no significant difference was found between the 1-month remineralization (1.68 ± 0.10) and sound dentine (1.65 ± 0.03) which were significantly higher.

## Discussion

Minimal-invasive dentistry involves the early diagnosis of carious lesions and a non-invasive treatment protocol [24]. With the preservation of the repairable CAD, this procedure involves the excavation of only the caries-infected dentine. In CAD, only partial demineralization occurs, so few apatite crystals remain attached to the collagen matrix even if the secondary structure of collagen appeared slightly altered when compared to that of sound dentine [36]. These apatite crystals act as nuclei for remineralization [36]. Though, it was discovered that the bond strength to CAD was weaker than that to sound dentine. This raised concerns regarding the CAD’s potential to remineralize, thus enhancing the bond strength.

The remineralizing agents used in this study were theobromine and CPP-ACPF having a trade name (MI paste plus) [13, 20, 37–40]. Theobromine of 200 mg/l was used as proposed by previous studies [13, 20] and reported as an effective method to remineralize enamel.

Two-time intervals were used; 5 min [19, 41] and one month [42, 43] in which the surface was covered for 1 month by a temporary restoration to simulate the clinical situation.

Considering the findings of our study, the null hypothesis was rejected as the surface morphology, microhardness, and resin-dentine bond strength of demineralized dentine changed as a result of the pre-treatment with both remineralizing agents as well as the various time intervals.

Regarding the impact of remineralizing agents on the µTBS, both theobromine and MI paste plus when applied for 5 min or 1 month showed µTBS values comparable to sound dentine and significantly higher than the demineralized dentine. This indicates that both materials irrelevant of the application times could restore the bond strength to values similar to that of sound dentine. These findings concur with those of Barbosa-Martins et al., [40] who stated in their study that the µTBS to CAD was much lower than sound dentine, and was restored after using CPP-ACP paste for 5 min.

The pre-treatment of demineralized dentine with theobromine for 5 min or 1 month increased the µTBS owing to its ability to remineralize demineralized tooth structure in an apatite-forming medium [22]. In a study by Amaechi et al., [22] theobromine has been shown to have a remineralization effect comparable to fluoride at a molar level (the amount of substance per unit volume of the solution) that is 71 times lower which means that less amount of theobromine is needed to induce the same remineralizing effect. The authors claimed that this resulted in increased crystallite size, and improved teeth crystallinity by growing HA in a medium capable of producing apatite that has adequate theobromine content. It was postulated that theobromine has a stimulatory effect on calcium and phosphorous to produce a crystal four times larger (2 μm) than HA (0.5 μm) [22, 44]. To ensure the availability of calcium and phosphorous needed for the remineralization process, the specimens were stored in SBF solution to simulate the fluid present in the pulp. Regarding the resin-dentin bond strength and durability, cacao seed extract is the most popular crosslinking agent [45]. In a study by Trier et al. [46] collagen fibril diameter in the posterior sclera was observed to be increased by theobromine.

CPP-ACPF pre-treatment of demineralized dentine for 5 min or 1 month also increased the µTBS. CPP binds with ACP, forming nano-complexes of CPP-ACP [17] stabilising calcium and phosphorous ion levels, and creating a condition of supersaturation around the teeth, which enhances remineralization of the teeth due to elevated ion and pH levels in the surrounding environment [16, 47]. Calcium and phosphate ions can easily permeate into the porous lesion, deposit in the partially demineralized crystals, and repair HA crystals [17, 48]. Studies have shown that CPP-ACP can promote dentine remineralization while reducing dentine demineralization [17, 48]. Behrouzi et al. [49] stated in their study that the remineralization process was accelerated by fluoride, calcium, and phosphorous ions penetrating the HA crystals, increasing their density. They are precipitated on the phosphorylated fibrils of the exposed intertubular dentine collagen [50].

Regarding microhardness, theobromine showed increased microhardness values when applied for 5 min or 1 month to demineralized dentine but still lower than sound dentine. This confirms its ability to remineralize the demineralized dentine surface. It had been suggested that theobromine might have penetrated the HA micro-tunnels and caused internal stress that made it harder to indent them thus increasing the microhardness [51].

Concerning the MI paste plus; its application for 5 min was insufficient to induce changes in the microhardness of the demineralized dentine whereas the 1-month application showed an increase in the microhardness values yet lower than sound dentine which indicates that MI paste plus required a longer period to remineralize the demineralized dentine surface.

SEM/EDX results match the results of the microhardness, MI paste plus produced changes in the Ca/P ratio on the dentine surface after 1 month that was evident in the SEM images where more mineral precipitates were observed on the dentine surface after a 1-month application. (Fig. 2)

When comparing the two materials, a significant difference was found between them at 5 min of application, where theobromine produced higher microhardness results. While after 1 month, there was no statistical difference between the two materials. These outcomes were consistent with Duraisamy et al. [19] who in their study compared the effect of theobromine 200 mg/l and CCP-ACPF when applied for 5 min on demineralized enamel, concluding that theobromine 200 mg/l has a higher remineralizing ability compared to CCP-ACPF. In a study by Mahesuti et al. [52] evaluating the effect of CPP-ACP in treating dentine hypersensitivity, they stated that it took the CPP-ACP 14 days to significantly produce an effect. In a study by Zhou et al. [26] microscopic analysis of the dentine morphology revealed that the dentine surface underwent a significant degree of remineralization after being exposed to CPP-ACP for 21 days, and the dentinal tubules were largely blocked. While in a study by Amaechi et al., [22] they stated that using a toothpaste containing theobromine demonstrated a rapid action in occluding dentinal tubules within the first day of use.

There was a controversy between the results of the µTBS and the microhardness, where the use of MI paste plus for 5 min was able to recover the µTBS to the sound dentine values but not the microhardness. This can be attributed to the use of Bisco all bond universal, whose functional monomer, 10-MDP, has an affinity to the HA crystals present around the demineralized collagen [53, 54]. An insoluble and resistant Ca-10-MDP monomer salt is formed as a result of this chemical reaction, which is supposed to improve the mechanical properties of bonding interfaces [36]. This might be why the µTBS was enhanced when using both remineralizing agents irrespective of the time but not the demineralized group as their use produced partial remineralization needed for the adhesive to give better adhesion.

Reviewing the literature regarding the correlation between the substrate microhardness and bond strength, Adebayo et al. [55] stated in their study that there was no correlation between micro-shear bond strength (µSBS) and enamel Vickers hardness number (VHN). Also, in a study by Bengtson et al. [56], the results showed no correlation between dentine nano-hardness and µTBS. In contrast, Wei et al. [57] found a significant correlation between dentine hardness and µSBS.

It is also noteworthy that given the short period of evaluation, it is infeasible to draw valid conclusions concerning the long-term success of these understudied remineralizing materials. While it has been claimed that studies with shorter observation periods might be of limited clinical relevance, short-time evaluations are indispensable for evaluating remineralizing materials. Also, aging procedures have major effects on the bond strength of the materials. Further investigations regarding the durability of the resin-dentine bond strength, and the effect of chewing simulation and thermocycling on bond strengths are suggested.

## Conclusions

Given the limitations of this in-vitro study, the following conclusions could be drawn:

1. The pre-treatment of demineralized dentine with theobromine for 5 min could enhance resin-dentine bond strength and microhardness.

2. Although 5 min of application of MI paste plus restored the resin-dentine bond strength, longer application times are needed to ensure a better remineralization effect.

## Data Availability

The datasets used and/or analysed during the current study are available from the corresponding author upon reasonable request.
